# Spicy Food and Chili Peppers and Multiple Health Outcomes: Umbrella Review

**DOI:** 10.1002/mnfr.202200167

**Published:** 2022-10-19

**Authors:** Zhimin Ao, Zongyue Huang, Hong Liu

**Affiliations:** ^1^ Department of Integrated Traditional and Western Medicine West China Hospital Sichuan University Chengdu 610041 China; ^2^ Department of Acupuncture and Moxibustion, The Sixth Medical Center of PLA General Hospital Department of Acupuncture and Moxibustion, The Sixth Medical Center of PLA General Hospital Beijing 100853 China

**Keywords:** capsicum consumption, health, meta‐analysis, spicy food consumption, uvmbrella review

## Abstract

Spicy foods and chili peppers contain the primary ingredient capsaicin, which has potential health benefits. However, their efficacy in some health outcomes is also fiercely disputed, and some side effects have been confirmed. To assess the quality and strength of the associations between spicy food and chili pepper consumption and different health outcomes. An umbrella review is performed in humans. Eleven systematic reviews and meta‐analyses with a total of 27 findings are identified. The health effect of consuming spicy food and chili peppers is unclear. Furthermore, the characteristics and context of different world regions and populations should be carefully considered. Direct correlations exist in esophageal cancer, gastric cancer, and gallbladder cancer. However, negative connections are reported in metabolism, mortality, and cardiovascular disease. Dose–response analysis reveals a significant nonlinear relationship between gastric cancer risk and capsaicin intake. The consumption of spicy foods and chili peppers is typically safe. However, high‐quality proof is available to confirm this conclusion.

## Graphical abstract

To assess the quality and strength of the associations between spicy food and chili pepper consumption and different health outcomes. An umbrella review is performed in humans. Direct correlations exist in esophageal cancer, gastric cancer, and gallbladder cancer. However, negative connections are reported in metabolism, mortality, and cardiovascular disease.

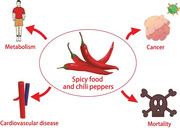

## 1 Introduction

Chili pepper (Solanaceae) has been valued since ancient times as a food crop,^[^ ^1^ ^]^ seasoning ingredient, natural dyestuff,^[^ ^2^ ^]^ and traditional herbal medicine^[^ ^3^ ^]^ and originated from approximately 9000 to 7000 B.P.^[^ ^4^ ^]^ In Mexico. Spicy food, the flavor and aroma of food created by the use of chili peppers, not only offers a significant hedonic input in daily living but also has been linked to health benefits. Chili peppers contain a diverse mix of phytochemicals, such as capsaicin, dihydrocapsaicin, total phenolic compounds, and antioxidant activity,^[^ ^5^ ^]^ which have broad therapeutic antimicrobial,^[^ ^6^ ^]^ antiseptic,^[^ ^3^ ^]^ antihypertensive,^[^ ^7^ ^]^ antioxidant,^[^ ^8^ ^]^ antiobesity,^[^ ^9^ ^]^ antihyperglycemic,^[^ ^10^ ^]^ and analgesic properties,^[^ ^11^ ^]^ protect against cardiometabolic vascular diseases,^[^ ^12^ ^]^ and have anticancer in vitro and animal studies.^[^ ^13^ ^]^ Capsaicin, available in various topical applications, is FDA‐approved for the treatment of diabetic peripheral neuropathic pain (DPNP)^[^ ^14^ ^]^ and neuropathic pain.^[^ ^15^ ^]^ Remain aware that excessive dosages of capsaicin may have unanticipated physiological effects due to the widespread expression of transient receptor potential vanilloid 1 (TRPV1) receptors.^[^ ^16^ ^]^

To date, the benefits and risks of spicy food and chili pepper consumption and their effect on health have been investigated by the majority of observational and epidemiological studies.^[^ ^17^ ^]^ However, there is no clear consensus in the literature about the effects of spicy foods and chili peppers on multiple health outcomes or no attempt to critically assess the quality and strength of the associations of spicy food and chili pepper consumption and different health outcomes. Chili pepper is one of the most commonly used condiments worldwide^[^ ^5^ ^]^; therefore, even potential minor effects could have profound implications for health at a population scale. Furthermore, substantial evidence exists to support the Mediterranean diet,^[^ ^18^ ^]^ but the spicy dietary pattern has less well‐established associations with health outcomes. Moreover, dietary characteristics vary around the world, the Mediterranean diet preference is not suitable for other nations, and we should expand our research into regional localization diet strategies. Finally, we should pay attention to the deficiency of research into the multitudinous health outcomes of dietary flavor.

This study set out to provide a comprehensive perspective of the evidence landscape of spicy food and chili peppers and multiple health outcomes. The umbrella review, only considering incorporation of the highest level of evidence, allows the results of relevant reviews for a research topic to be compared and evaluated.^[^ ^19^ ^]^ To the best of our knowledge, no umbrella review evaluates the validity and breadth of evidence available about spicy food and chili pepper consumption to all health outcomes in humans. Thus, we performed this study to address the problem and evolve our understanding of the effects that various dietary taste patterns and other food components play on health and disease.

## 2 Results

### 2.1 Characteristics of the Study

**Figure 1:**
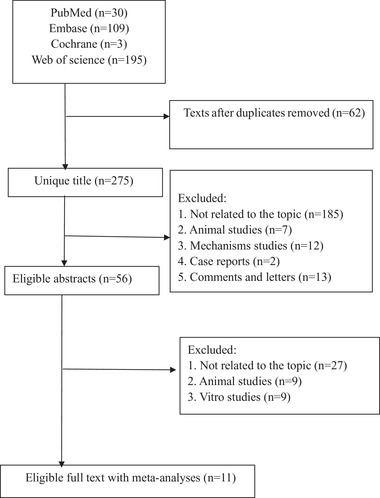
Flowchart of the selection process.

**Figure 2:**
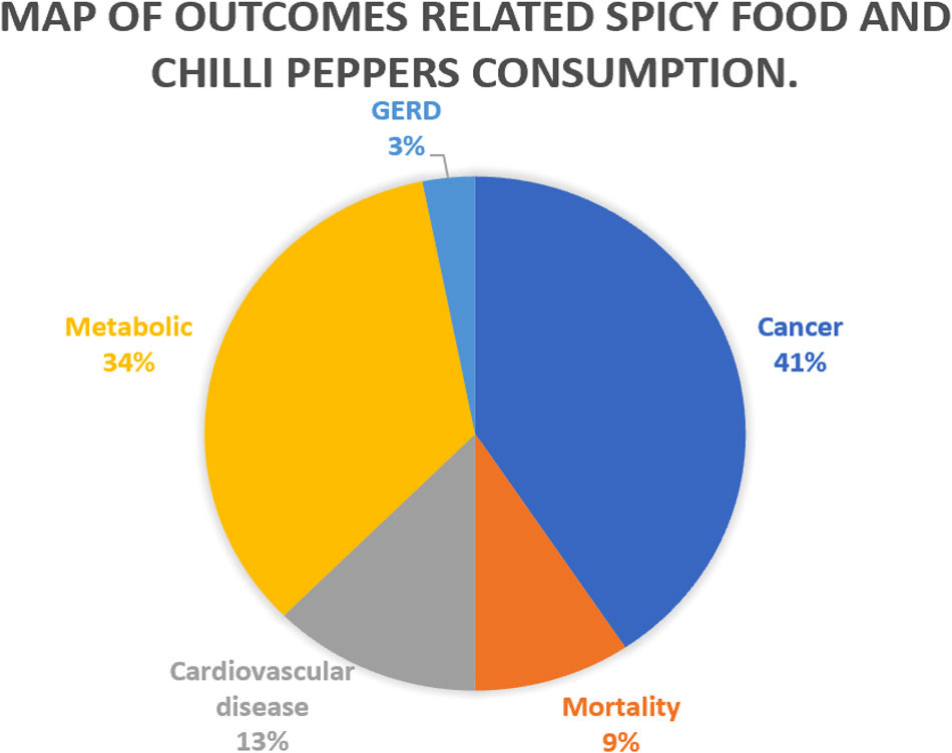
Map of outcomes related to spicy food and chili pepper consumption.

**Figure 3:**
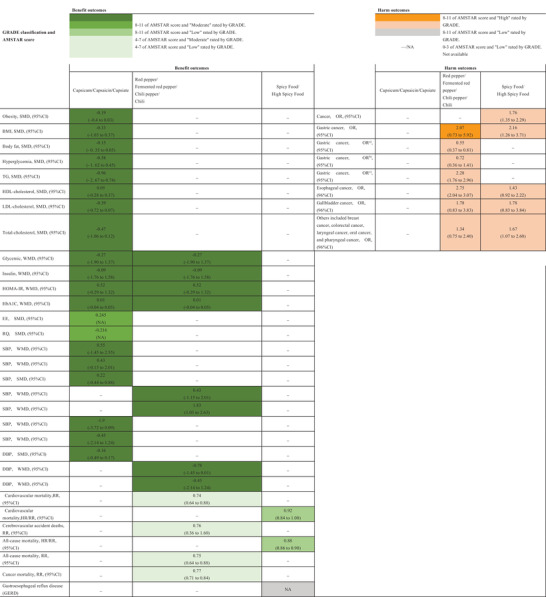
Summary of AMSTAR and GRADE classification of spicy food and chili pepper consumption on benefit and harm outcomes.

The flow diagram of the study selection process is shown in **Figure** **1**. A total of 275 articles existed in our initial search. Following the application of the predefined inclusion and exclusion criteria, 11 articles ultimately met our inclusion criteria. **Figure** **2** illustrates a map of health outcomes linked to spicy food and chili pepper intake, which includes 11 systematic reviews with 27 outcomes. **Figure** **3** shows the summary of AMSTAR and GRADE Classification of spicy food and chili pepper consumption on benefit and harm outcomes. **Tables** **1–3** show the associations between spicy food and chili pepper intake and health outcomes. The assessments of AMSTAR scores and GRADE classification are presented in **Table** **4**.

**Table 1:** Associations between spicy food and chili pepper consumption and metabolic diseases

Outcome	Category	Study	No. of cases/total	MA metric	Estimates	95% CI	No. of studies in MA	Cohort	Case control	Cross‐sectional	Effects model	*I* ^2^	Egger test *p*‐value
Obesity	Capsicum	Jang 2020	NA	SMD	−0.19	−0.4–0.03	7	NA	NA	NA	Random	0%	NA
BMI	Capsicum	Jang 2020	NA	SMD	−0.33	−1.03–0.37	5	NA	NA	NA	Random	85%	NA
Body fat	Capsicum	Jang 2020	NA	SMD	−0.15	−0.35–0.05	9	NA	NA	NA	Random	18%	NA
Hyperglycemia	Capsicum	Jang 2020	NA	SMD	−0.58	−1.62–0.45	3	NA	NA	NA	Random	91%	NA
TG	Capsicum	Jang 2020	NA	SMD	−0.96	−2.67–0.74	3	NA	NA	NA	Random	95%	NA
HDL‐cholesterol	Capsicum	Jang 2020	NA	SMD	0.05	−0.28–0.37	3	NA	NA	NA	Random	0%	NA
LDL‐cholesterol	Capsicum	Jang 2020	NA	SMD	−0.39	−0.72–0.07	3	NA	NA	NA	Random	13%	NA
Total‐cholesterol	Capsicum	Jang 2020	NA	SMD	−0.47	−1.06–0.12	3	NA	NA	NA	Random	71%	NA
Glycemic	Capsinoids and fermented red pepper	Reza Amini 2021	NA/530	WMD	−0.27	−1.90–1.37	8	NA	NA	NA	Random	59.60%	0.297
Insulin	Capsinoids and fermented red pepper	Reza Amini 2021	NA/530	WMD	−0.09	−1.76–1.57	4	NA	NA	NA	Random	77.20%	0.076
HOMA‐IR	Capsinoids and fermented red pepper	Reza Amini 2021	NA/530	WMD	0.52	−0.29–1.32	4	NA	NA	NA	Random	91.50%	0.076
HbA1C	Capsinoids and fermented red pepper	Reza Amini 2021	NA/530	WMD	0.01	−0.04–0.05	3	NA	NA	NA	Random	NA	0.955
EE	Capsaicin and capsiate	Zsiborás 2018	NA/255	SMD	0.245	NA	9	NA	NA	NA	Fixed	NA	NA
RQ	Capsaicin and capsiate	Zsiborás 2018	NA/256	SMD	−0.216	NA	9	NA	NA	NA	Fixed	NA	NA
EE	Capsaicin	Ludy 2012	NA/255	SMD	0.11	−0.06–0.29	7	NA	NA	NA	Fixed	16.60%	NA
RQ	Capsaicin	Ludy 2012	NA/255	SMD	−0.35	−0.54–0.15	10	NA	NA	NA	Fixed	6%	NA
EE	Capsiate	Ludy 2012	NA/255	SMD	0.4	0.22–0.59	13	NA	NA	NA	Fixed	0%	NA
RQ	Capsiate	Ludy 2012	NA/255	SMD	−0.31	−0.54–0.07	9	NA	NA	NA	Fixed	26.80%	NA

BMI, body mass index; EE, energy expenditure; HbA1C, hemoglobin A1C; HOMA‐IR, homeostasis model assessment of insulin resistance; NA, not available; RQ, respiratory quotient; SMD, standard mean difference; TG, triacylglycerol; WMD, weighted mean difference.

### 2.2 Cancer Outcomes

Spicy food and pepper intake is positively related to various cancers: esophageal cancer,^[^ ^20^ ^]^ gastric cancer,^[^ ^21^ ^]^ gallbladder cancer,^[^ ^20^ ^]^ and other cancers, including breast cancer, colorectal cancer, laryngeal cancer, oral cancer, and pharyngeal cancer.^[^ ^20^ ^]^ Moderate to high pepper consumption in the Asian population was significantly linked to gastric cancer in Table 3 (OR = 2.24, 95% CI 1.88–2.67, *p* = 0.005); however, low pepper consumption did not appear to be related to an increased risk of gastric cancer in Table 3 (OR = 0.62, 95% CI 0.33–1.18, *p* = 0.144). Dose–response analysis found a significant nonlinear relationship between GC risk and capsaicin intake (*p* nonlinearity <0.05).^[^ ^22^ ^]^

### 2.3 Mortality

Chili‐pepper consumption could reduce the risk of all‐cause mortality with a risk ratio (RR) of 0.75 [95% CI: 0.64–0.88] compared to nonconsumers as well as a lower risk of cancer mortality moderate to high pepper consumption in the Asian population was significantly linked to gastric cancer in Table 2 (RR: 0.77; 95% CI: 0.71–0.84).^[^ ^23^ ^]^ The consumption of spicy food is associated with a 12% lower risk of all‐cause mortality in Table 2 (HR/RR = 0.88, 95% CI, 0.86–0.90; *I* ^2^ = 0%).^[^ ^24^ ^]^

**Table 2:** Associations between spicy food and chili pepper consumption and cardiovascular and mortality diseases

Outcome	Category	Study	No. of cases/total	MA metric	Estimates	95% CI	No. of studies in MA	Cohort	Case control	Cross‐sectional	Effects model	*I* ^2^	Egger test *p*‐value
**Cardiovascular outcomes**													
DBP	Capsicum	Jang 2020	NA	SMD	−0.16	−0.49–0.17	3	NA	NA	NA	Random	0%	NA
SBP	Capsicum	Jang 2020	NA	SMD	0.22	−0.44–0.88	4	NA	NA	NA	Random	81%	NA
SBP	Red pepper/Capsaicin	Shirani 2021	NA	WMD	0.43	−1.15–2.01	8	NA	NA	NA	Random	72.50%	0.267
DBP	Red pepper/Capsaicin	Shirani 2021	NA	WMD	−0.45	−2.14–1.24	7	NA	NA	NA	Random	74.70%	0.959
Heart rate	Red pepper/Capsaicin	Shirani 2021	NA	WMD	−0.6	−1.97–0.78	5	NA	NA	NA	Random	46.80%	0.001
SBP	Fermented red pepper	Reza Amini 2020	NA/363	WMD	1.83	1.03–2.63	8	NA	NA	NA	Random	54.30%	0.228
DBP	Fermented red pepper	Reza Amini 2020	NA/363	WMD	−0.78	−1.45–0.01	7	NA	NA	NA	Random	66.50%	0.409
SBP	Capsinoids	Reza Amini 2020	NA/363	WMD	0.55	−1.45–2.55	8	NA	NA	NA	Random	54.30%	0.228
DBP	Capsinoids	Reza Amini 2020	NA/363	WMD	−1.9	−3.72–0.09	7	NA	NA	NA	Random	66.50%	0.409
Cardiovascular mortality	Chili pepper	Yamani 2021	NA/570 762	RR	0.74	0.64–0.88	4	4	0	0	Random	66%	NA
Cerebrovascular accident deaths	Chili pepper	Yamani 2022	NA/570 763	RR	0.76	0.36–1.60	4	4	0	0	Random	93%	NA
Cardiovascular mortality	Spicy food	Ofori‐Asenso 2021	NA/564 748	HR/RR	0.92	0.84–1.00	4	4	0	0	Random	0%	NA
**Mortality**													NA
All‐cause mortality	Chili‐pepper	Yamani 2021	NA/570 762	RR	0.75	0.64–0.88	4	4	0	0	Random	97%	NA
Cancer mortality	Chili‐pepper	Yamani 2021	NA/570 762	RR	0.77	0.71; 0.84	4	4	0	0	Random	49%	NA
All‐cause mortality	Spicy food	Ofori‐Asenso 2021	NA/564 748	HR/RR	0.88	0.86–0.90	4	4	0	0	Random	0%	NA

DBP, diastolic blood pressure; HR, hazard ratio; NA, not available; RR, relative risk; SBP, systolic blood pressure; SMD, standard mean difference; WMD, weighted mean difference.

### 2.4 Cardiovascular Disease

Spicy food and pepper intake are associated with significant decreases in cardiovascular mortality^[^ ^25^ ^]^ and cerebrovascular accident deaths in Table 2.^[^ ^23^ ^]^ Furthermore, fermented red pepper supplementation may play a part in improving systolic blood pressure (SBP) and diastolic blood pressure (DBP).^[^ ^26^ ^]^ However, there was no significant association between the intake of red pepper/capsaicin and SBP, DBP, and heart rate.^[^ ^27^ ^]^

### 2.5 Metabolic Outcome

Evidence has shown a beneficial effect of capsicum annuum supplementation on body weight [SMD = −0.19; 95% CI −0.40, 0.03], low density lipoprotein‑cholesterol in Table 1.^[^ ^28^ ^]^ However, there was no significant association among triacylglycerol (TG), HDL‐cholesterol, total cholesterol,^[^ ^28^ ^]^ blood glucose, insulin, homeostasis model assessment of insulin resistance (HOMA‐IR), and hemoglobin A1C (HbA1C) in Table 1.^[^ ^29^ ^]^

### 2.6 Other Outcomes

There is a lack of sufficient evidence to forbid eating spicy food for the prevention or treatment of gastroesophageal reflux disease (GERD) in Table 3.^[^ ^26^ ^]^

**Table 3:** Associations between spicy food and chili pepper consumption and cancer outcomes and GERD

Outcome	Category	Study	No. of cases/total	MA metric	Estimates	95% CI	No. of studies in MA	Cohort	Case control	Cross‐sectional	Effects model	*I* ^2^	Egger test *p*‐value
**Cancer outcomes**													
Cancer	High spicy food	Chen 2016	7884/18 026	OR	1.76	1.35–2.29	39	0	39	0	Random	88.30%	0.714
Gastric cancer	High spicy food	Chen 2017	NA/18 026	OR	2.16	1.26–3.71	12	0	12	0	Random	91.30%	NA
Gastric cancer	Chili	Chen 2017	NA/18 026	OR	2.07	0.73–5.91	6	0	6	0	Random	94.00%	NA
Esophageal cancer	High spicy food	Chen 2017	NA/18 026	OR	1.43	0.92–2.22	9	0	9	0	Random	77.10%	NA
Esophageal cancer	Chili	Chen 2017	NA/18 026	OR	2.75	2.04–3.70	4	0	4	0	Random	9.60%	NA
Gallbladder cancer	High spicy food	Chen 2017	NA/18 026	OR	1.78	0.83–3.83	6	0	6	0	Random	75.00%	NA
Gallbladder cancer	Chili	Chen 2017	NA/18 026	OR	1.78	0.83–3.83	6	0	6	0	Random	75.00%	NA
Others included breast cancer, colorectal cancer, laryngeal cancer, oral cancer, and pharyngeal cancer.	High spicy food	Chen 2017	NA/18 026	OR	1.67	1.07–2.60	12	0	12	0	Random	90.00%	NA
Others included breast cancer, colorectal cancer, laryngeal cancer, oral cancer, and pharyngeal cancer.	Chili	Chen 2017	NA/18 026	OR	1.34	0.75–2.40	7	0	7	0	Random	91.70%	NA
Gastric cancer	Chili	Du 2020	3095/7856	OR	1.96	1.59–2.42	13	0	13	0	Random	74.70%	0.288
Gastric cancer	Chili	Du 2020	NA/7856	OR^a)^	0.55	0.37–0.81	NA	NA	NA	NA	Random	NA	NA
Gastric cancer	Chili	Du 2020	NA/7857	OR^b)^	0.72	0.36–1.41	NA	NA	NA	NA	Random	NA	NA
Gastric cancer	Chili	Du 2020	NA/7858	OR^c)^	2.28	1.76–2.96	NA	NA	NA	NA	Random	NA	NA
GERD	Spicy food	Castillo 2015	NA	NA	NA	NA	NA	NA	NA	NA	NA	NA	NA

GERD, gastroesophageal reflux disease; NA, not available; OR, odds ratio;

^a)^Low consumption (0–30 mg day^‐1^);

^b)^Moderate consumption (30–90 mg day^‐1^);

^c)^High consumption (90–250 mg day^‐1^).

### 2.7 Side Effects

The spicy food and chili pepper consumption reported no serious adverse effects. However, some studies reported adverse events, including leg cramps,^[^ ^30^ ^]^ bowel irregularities, skin rash,^[^ ^31^ ^]^ heat sensation in the oral cavity1, and skin wheals.^[^ ^32^ ^]^

### 2.8 Heterogeneity

Six (12.5%) outcomes demonstrated low levels of heterogeneity (*I* ^2^ < 25%), 16 (33%) showed moderate‐to‐high levels of heterogeneity (*I* ^2^ = 25–75%), 13 (27%) indicated extremely high heterogeneity (*I* ^2^ > 75%), seven showed little heterogeneity (*I* ^2^ = 0%), and six (22.2%) did not reported heterogeneity.

### 2.9 GRADE Classification and AMSTAR Score

Overall, three (27.2%) of evidence were classified as “very low” or “low” level of qualities by GRADE, seven (64%) as “moderate” and one (9.0%) as “high” level of the quality. The mean AMSTAR score was 9.32 (range 3.0–11.0). The detailed AMSTAR scores and GRADE classifications for each study are presented in Table 4.

**Table 4:** Assessments of AMSTAR scores and GRADE classification

Outcome	Category	Author	Year	AMSTAR	GRADE
Obesity	Capsicum	Jang	2020	10	Moderate
BMI	Capsicum	Jang	2020	10	Moderate
Hypertension: diastolic blood pressure (DBP)	Capsicum	Jang	2020	10	Moderate
Hypertension: systolic blood pressure (SBP)	Capsicum	Jang	2020	10	Moderate
Dyslipidemia: hyperglycemia	Capsicum	Jang	2020	10	Moderate
Dyslipidemia: triacylglycerol (TG)	Capsicum	Jang	2020	10	Moderate
Dyslipidemia: HDL‐cholesterol	Capsicum	Jang	2020	10	Moderate
Dyslipidemia: LDL‐cholesterol	Capsicum	Jang	2020	10	Moderate
Dyslipidemia: Total‐cholesterol	Capsicum	Jang	2020	10	Moderate
Insulin	Capsinoids and fermented red pepper	Reza Amini	2021	11	Moderate
HOMA‐IR, homeostasis model assessment of insulin resistance	Capsinoids and fermented red pepper	Reza Amini	2021	11	Moderate
Hemoglobin A1C (HbA1C)	Capsinoids and fermented red pepper	Reza Amini	2021	11	Moderate
Blood sugar	Capsinoids and fermented red pepper	Reza Amini	2021	11	Moderate
Hypertension: systolic blood pressure (SBP)	Red pepper/Capsaicin	Shirani	2021	11	Moderate
Hypertension: diastolic blood pressure (DBP)	Red pepper/Capsaicin	Shirani	2021	11	Moderate
Heart rate	Red pepper/Capsaicin	Shirani	2021	11	Moderate
Hypertension: systolic blood pressure (SBP)	Fermented red pepper	Reza Amini	2021	11	Moderate
Hypertension: diastolic blood pressure (DBP)	Fermented red pepper	Reza Amini	2021	11	Moderate
Energy expenditure, EE	Capsaicin and capsiate	Zsiborás	2018	10	Moderate
Respiratory quotient, RQ	Capsaicin and capsiate	Zsiborás	2018	10	Moderate
Energy expenditure, EE	Capsaicin	Ludy	2012	5	Moderate
Respiratory quotient, RQ	Capsaicin	Ludy	2012	5	Moderate
Gastric cancer	High Spicy Food	Chen	2017	11	Low
Esophageal cancer	High Spicy Food	Chen	2017	11	Low
Gallbladder cancer	High Spicy Food	Chen	2017	11	Low
Others included breast cancer, colorectal cancer, laryngeal cancer, oral cancer, and pharyngeal cancer.	High Spicy Food	Chen	2017	11	Low
Gastric cancer	Chili	Du	2020	11	High
All‐cause mortality	Chili‐pepper	Yamani	2021	5	Low
Cardiovascular mortality	Chili‐pepper	Yamani	2021	5	Low
Cancer mortality	Chili‐pepper	Yamani	2021	5	Low
Cerebrovascular accident deaths	Chili‐pepper	Yamani	2021	5	Low
All‐cause mortality	Spicy food	Ofori‐Asenso	2021	10	Low
Cardiovascular mortality	Spicy food	Ofori‐Asenso	2021	10	Low
Gastroesophageal reflux disease (GERD)	Spicy food	Castillo	2015	3	Very low

## 3 Discussion

### 3.1 Main Findings and Interpretation

We examined 11 systematic reviews in the umbrella review to evaluate the relationship between spicy food and chili pepper consumption and health outcomes. Overall, the health effects of consuming spicy foods and chili peppers are uncertain. The following outcomes had a direct correlation with spicy food and capsicum intake: esophageal cancer, gastric cancer, or gallbladder cancer. However, there was a negative correlation between the intake of spicy food and capsicum and blood pressure, energy expenditure, respiratory quotient, obesity, all‐cause mortality, cardiovascular mortality, or cerebrovascular accident deaths. No significant association exists in blood glucose, insulin, homeostasis model assessment of insulin resistance (HOMA‐IR), hemoglobin A1C (HbA1C), or gastroesophageal reflux disease (GERD). A significant nonlinear relationship between GC risk and capsaicin intake revealed by dose–response analysis. GRADE classified over half (64%) of the evidence quality as “moderate.”

Capsaicin and spicy food consumption and metabolism were some of the most apparent links found in this study. Growing evidence indicates that capsaicin and spicy food consumption favor metabolism. Further studies on metabolic syndrome indicated that capsaicin is a potent dietary supplement in the treatment of obesity and insulin resistance.^[^ ^33^ ^]^ On the other hand, capsaicin has also been shown to manifest antihyperglycemic and antiobesity effects by modulating the gut‐brain axis and inhibiting the enterohepatic FXR‐FGF15 axis.^[^ ^34^ ^]^ Recent findings about the association between capsaicin and gut microbiota composition, abundance, and function have reported the possible mechanisms by which capsaicin exerts its influence.^[^ ^34^ ^]^ An epidemiological study announced that the consumption of capsaicin‐containing foods is connected with a lower prevalence of obesity.^[^ ^35^ ^]^ Rural Thais consume food containing 0.014% capsaicin. Rodents administered a diet containing 0.014% capsaicin showed no difference in calorie consumption, although there was a significant decline in visceral (perirenal) fat weight of 24%^[^ ^36^ ^]^ and 29%.^[^ ^37^ ^]^ This evidence suggests that in general, capsaicin and spicy foods may provide fresh insight into the prevention and management of metabolic syndrome. However, when confronted with different world regions and populations, this conclusion requires more caution in its application. Because of the factors contributing to MetS, such as genetic, environmental, and lifestyle factors, the risk of metabolic syndrome (MetS) varies significantly between populations and world regions.^[^ ^38^ ^]^ According to some research, the gut microbiome may be the genesis of the pathways leading to MetS risk factors.^[^ ^39^, ^40^ ^]^ The constitution and behavior of gut microbiome from different populations could exert an effect on the emergence of low‐grade inflammation,^[^ ^41^ ^]^ insulin resistance, obesity, and dyslipidemia.^[^ ^42^ ^]^ This was clearly evidenced in Martha's study.^[^ ^39^ ^]^ Furthermore, additional discussion should be made of different world regions and populations the unique metabolic digestion of nutrients, genetics, lifestyle habits.

Research applying the national data of the China Health and Nutrition Survey (CHNS) demonstrated that even though individuals have the same preference for spicy foods, different food consumption will have different metabolic impacts.^[^ ^43^ ^]^ The spicy food preference is more of a psychological attitude‐correlated choice than a behavior, so it is not clear what effect it has on the metabolism. For example, intakes of dietary sugars make several contributions to Mets, Chinese children consumed 26 g d^−1^ of total and added sugars, Mexican children 92 g d^−1^, and US children 124 g d^−1^.^[^ ^44^ ^]^ These data indicated that we were able to evaluate the food categories of daily intake across nations, thereby establishing the interaction between dietary preferences and health. Hypertension and Mets are interrelated and co‐occurrence,^[^ ^45^ ^]^ the incidence changes in each country that was due to changes in population size, age composition, and age‐specific prevalence under various world regions and populations. For instance, changing demographics alone will increase the number of individuals in need of care for hypertension by 319.7 million, varying from a relative increase of 55% in China to a relative increase of 151% in Mexico.^[^ ^46^ ^]^

The results in this review showed no significant association among triacylglycerol (TG), HDL‐cholesterol, total cholesterol, blood glucose, and insulin.^[^ ^28^, ^30^ ^]^ Moreover, when used in the medical field, it necessitates strict adherence to a certain dosage, which has not been proven to be practical.^[^ ^47^ ^]^ It is possible, therefore, that the results are affected by some restrictions (DNA, habitat, climate, lifestyle, and energy expenditure).^[^ ^48^ ^]^ A range of Asian‐based diets contain spicy food compared with Western countries; similarly, the prevalence of obesity is as low as 3%, but it is well over 10% in most Western countries.^[^ ^49^ ^]^ A large‐scale nationwide Internet data‐based study in China including 212 314 708 individuals reported that dietary preferences ranged by geographical distribution, with higher altitude regions covering large proportions of spicy food; likewise, spicy food appetite was inversely connected to diabetes risk.^[^ ^50^ ^]^ Further studies are needed to clarify the interaction between dietary preferences and environmental health. Some people prefer spicy flavors based on a demand to release pressure and stimulate appetite, which is accompanied by an increase in energy intake. Therefore, in the case of consistent energy intake and food intake, adopting a spicy diet will bring certain metabolic benefits.

Our umbrella review found sufficient evidence that capsaicin and spicy food consumption were also positively correlated with cancer outcomes. Some evidence presented thus far supports the idea that a study reported that topical capsaicin treatment caused more skin papillomas in TRPV1 knockout mice than in TRPV1 wild‐type animals.^[^ ^51^ ^]^ Then, capsaicin induced EGFR‐tyrosine phosphorylation. These findings suggest that capsaicin might act as a cocarcinogen in a TRPV1‐independent but EGFR‐dependent manner.^[^ ^52^ ^]^ Furthermore, capsaicin has also been reported to boost the proliferation and survival of androgen‐responsive prostate cancer LNCaP cells, which corresponds to enhanced androgen receptor expression.^[^ ^53^ ^]^

However, research data contradictory to the results in this review showed that capsaicin can serve as a cancer resistance agent. First, TRPV1 is a possible treatment for a wide range of illnesses, such as inflammation, cancer, and autoimmune diseases. Some studies are increasingly turning attention to the association between TRPV1 activation by capsaicin and anticancer effects.^[^ ^54^ ^]^ Second, in many studies, capsaicin's anticancer activity against various cancers has been established to delay tumor progression, limit metastasis, and improve survival rates.^[^ ^55^ ^]^ Finally, according to mouse models of HCC, CCA, pancreatic cancer, and colorectal cancer, capsaicin inhibits the development of cancer cells in vivo by acting on cancer‐related genes and signaling pathways.^[^ ^56^ ^]^ Conflicting evidence offers further support for the hypothesis that the ultimate effects of capsaicin may be based on the dosage. Furthermore, capsaicin seems to have a biphasic function, supporting growth at low dosages and causing apoptosis at concentrations greater than 200 µM.^[^ ^57^ ^]^ In addition, the contamination of spicy foods with carcinogenic chemicals such as aflatoxin complicates their interpretation.

Another association identified in our umbrella review was about cardiovascular outcomes. TRPV1 possesses multiple mechanisms for cardiovascular protection. Capsaicin induces subsequent physiological processes by activating TRPV1. Moreover, TRPV1 can play a potential role in protecting cardiac metabolism, implying that TRPV1 may be a promising target for the treatment of cardiac metabolic diseases.^[^ ^58^, ^59^ ^]^

### 3.2 Safety

People who suffer from gastrointestinal discomfort should limit the intake of spicy food. Other physiological effects, such as neurotoxicity, redness, and allergies, result in high doses of capsaicin consumption.^[^ ^60^ ^]^

### 3.3 Strengths and Limitations

Umbrella reviews, only considering incorporation of the highest level of evidence, are studies that identify, assess, and synthesize the outcomes of diverse research on a certain issue, resulting in credible data in a useable format. The associations between capsaicin and spicy food consumption and various health outcomes evaluated in this review comprehensively and systematically. However, there are some potential limitations that should be emphasized in this review. First, the evidence has a low level of strength, and almost 43.7% of GRADE's evidence had a “very low” or “low” level of quality. Second, we cannot address this relationship between chili peppers and spicy food intake and health outcomes going further because of the lack of dosage response analysis. Finally, some important but not in accordance with the inclusion criteria studies might be missed, such as valuable animal experimental studies.

## 4 Conclusions

In conclusion, the effect of capsaicin and spicy food consumption to a range of health outcomes in humans is less clear. The evidence, moreover, was not of extremely high quality. In addition, high‐quality researches are further required to appraise the validity of evidence. Notwithstanding these limitations, this study offers comprehensive insights into spicy food and chili peppers and multiple health outcomes.

## 5 Experimental Section

### Umbrella Review Methods and Literature Search

This umbrella review thoroughly searched and analyzed available evidence from systematic reviews and meta‐analyses of multiple healthy outcomes associated with spicy food and capsicum consumption. Four databases, including PubMed, Web of Science, the Cochrane Database, and Embase, were searched from inception until November 9, 2021. The following search strategy was used: (capsicum) AND (systematic review* or meta‐analys*). The study also searched references from all eligible articles. Any disagreement was resolved by discussion with the authors.

### Eligibility Criteria

Systematic reviews assessing the relevance of capsicum and spicy food and multiple health outcomes were included. Chinese and English were not limited. If there was more than one similar article, only the latest could be included. The study excluded articles such as animal, in vitro, research mechanism studies, comments, and letters.

### Data Extraction

The following data were extracted by two authors: 1) outcomes involved any types of health in humans, 2) type of spicy food and chili peppers, 3) dosage and frequency of spicy food and chili peppers, 4) the first author, journal, 5) publication year, 6) number of total studies, 7) number of total participants, 8) the kind of study design, 9) the type of effect model, 10) relative estimated effect (OR, RR, SMD, WMD, HR, HR/RR), 11) the 95% confidence intervals (CIs), 12) heterogeneity, 13) publication bias, and 14) dose–response analyses. Any disagreement was resolved by discussion with the authors.

### Evidence Evaluation and Grading

The methodological quality was assessed by two review authors independently using the AMSTAR checklist with 11 elements.^[^ ^61^ ^]^ Authors performed GRADE to assess the strength of the evidence.^[^ ^62^ ^]^

### Data Analysis

The study extracted the estimated summary effect and the 95% CI from every published systematic review. The heterogeneity among studies was evaluated by Cochran's *Q* test and the *I* ^2^ metric. The random effects methods were taken into account if the *I* ^2^ statistic was high over 50%. Egger's method was conducted to calculate publication bias, with a *p* value compared with fewer than 0.1 judged significant for small‐study effects and heterogeneity. Otherwise, the significance criterion was set to *p* < 0.05. Dose–response analyses were abstracted when data were available.

## Conflict of Interest

The authors declare no conflict of interest.

## Author Contributions

Z.A. and H.L. designed the study. Z.A., H.L, and Z.H. conducted data extraction and analysis. Z.A. wrote the manuscript. Any disagreement was resolved by discussion with the authors (Z.A., Z.H., and H.L.). The manuscript was reviewed and approved by all authors.
